# Potential protective and therapeutic role of immune checkpoint inhibitors against viral infections and COVID-19

**DOI:** 10.2217/imt-2020-0109

**Published:** 2020-06-29

**Authors:** Lidia Gatto, Enrico Franceschi, Vincenzo Di Nunno, Alba Ariela Brandes

**Affiliations:** ^1^Department of Medical Oncology, Azienda USL/IRCCS Institute of Neurological Sciences, Bologna, Italy

**Keywords:** cancer immunotherapy, COVID-19, cytokines storm, immune checkpoint inhibitors, SARS-CoV-2, viral infections

## Cancer care during COVID-19 pandemic

On 11 March 2020, the WHO declared the 2019 novel coronavirus outbreak an international Public Health Emergency [1]. Cancer patients represent a particularly vulnerable population, at increased risk of COVID-19 with a worse outcome than individuals without cancer [2]. Since the US FDA approval of the CTLA-4 inhibitor ipilimumab in 2011, indications for immune checkpoint inhibitors (ICI) have dramatically expanded, becoming an effective treatment option for a wide variety of cancers, including melanoma, renal carcinoma, lung cancer, urothelial cancer and head and neck carcinoma. It is still unclear how to manage checkpoint inhibitor therapy in the context of the current emergency, due to the alleged alert of serious pneumonia in case of COVID-19 [3]. Human COVID-19 presents with a wide spectrum of clinical manifestations ranging from asymptomatic to severe disease. Respiratory epithelial cells are the primary targets for the virus and are a leading source of immunopathological injury.

HLA host haplotype are fundamentally important. Clinically, the immune response induced by COVID-19 is two-phased. First, the incubation period, a nonsevere stage, during which the virus induces a protective immune response.

In this phase strategies to boost immune response (antisera or pegylated IFN-α) are adopted, aimed at eliminating the virus and avoiding disease progression to severe stages. For the development of an endogenous protective immune response, a good general health status and an appropriate HLA host haplotype are fundamental.

Different HLA haplotypes contribute to individual variations in defense against pathogens and are associated with distinct susceptibility to various infectious diseases. Accordingly, it seems to be advantageous to have specific HLA haplotype with increased binding specificities to the SARS-CoV-2 peptides. When the protective immune response is inadequate, the patient enters the second phase, the severe stage, characterized by strong inflammatory response, massive tissue damage especially in the lung and in organs that have high angiotensin-converting enzyme 2 expression, such as intestine and kidney [4]. The severe cases present with pneumonia which can lead to diffuse alveolar and capillary injury, pulmonary edema and acute respiratory distress syndrome, a condition that can rapidly evolve in a multiorgan failure. The pathophysiology of unusually high pathogenicity for COVID-19 has not been completely understood; one of the supposed mechanisms underlying disease severity is a hyperinduction of pro-inflammatory cytokines, known as ‘cytokine storm’ [5]. It is a vasculitic-like condition that resembles the secondary hemophagocytic lymphohistiocytosis, characterized by increased levels of IL-2, IL-7,G-CSF, IFN-γ inducible protein 10, monocyte chemoattractant protein 1, macrophage inflammatory protein 1-α (MIP1A) and TNF-α. Similar to the SARS disease, the main histological finding in COVID-19 is an exudative and proliferative lung injury, with the formation of a clear liquid jelly and hyaline membrane. Tissue-resident macrophages overactivation is implicated in the release of a storm of cytokines leading to multiorgan dysfunction, such as pancytopenia, disseminated intravascular coagulation, hepatobiliary and central nervous system dysfunction. The crux of the pathogenesis is the overproduction, by tissues macrophages, of IL-1b, which drives the acute phase response to infection, the Th17 differentiation and the immunopathogenic response observed in acute respiratory distress syndrome and COVID-19 [6]. IL-1b and TNF-α both induce TH17 cell responses and vascular permeability. The overactivation of T cells, manifested by an increase of Th17 and high cytotoxicity of CD8 T cells, accounts for, in part, the severe immune injury. TH17 cells produce IL-17 and GM-CSF, both associated with autoimmune and inflammatory disease. IL-17 promotes the production of G-CSF, IL-6, IL-1b and TNF-α that lead to systemic inflammatory symptoms, including fever. Chemokines like MIP2A, IL-8, IP10, MIP3A engage immune infiltrates [7]. IFN-γ is pleiotropic cytokine that enhances IL-6 production in monocytes and activates a large downstream cascade through the Janus kinase 1/Janus kinase 2 signaling. IL-6 promotes recruitment of neutrophils and cytotoxic T cells and has a crucial role: it inhibits the ability of lung dendritic cells to trigger naive T cells, thereby minimizing the adaptive immune response [6]. Huang *et al.* have performed comparison between patients who had been admitted to the intensive care unit and those who had not, observing that serum levels of IL2, IL7, IL10,G-CSF, IP10, MCP1, MIP1A and TNF-α are higher in intensive care units patients, suggesting that the cytokine storm is associated with disease severity [8]. Cancer immunotherapy works by reducing the suppression of the effector T cells, respiratory epithelial cells especially CD8^+^, thereby restoring the cellular immunocompetence and improving tumor-specific immune responses. The main adverse effects of immune checkpoints inhibitors are related to excessive production of pro-inflammatory cytokines IL-6 and TNF-α; the immune-mediated lung injury, termed checkpoint inhibitor pneumonitis, occurs in about 3–5% of patients receiving ICI; however, the real-world incidence may be higher, especially now that ICI are used outward of clinical trials [9]. Thus, a potential synergy between the lung toxicity from ICI and the SARS-CoV-2 related interstitial-pneumonia has been hypothesized in patients receiving anticancer immunotherapy [3]. Patients receiving ICI are in a hyperimmune condition and can develop aberrant reactions in case of COVID-19 infection due to the overlap between the two pathogenetic mechanisms of lung injury. Is it possible to estimate the risk of contracting infections of cancer patients treated with immunotherapy compared with patients treated with chemotherapy and compared with the general population? Although our observations have no epidemiological background, we are quite surprised by the limited number of patients undergoing immunotherapy contacting us due to COVID-19.

## Role of ICI against viral infections & SARS-CoV-2

ICIs are currently widely used in oncology due to dramatic and durable responses in a variety of tumor types, but there are few data on their possible use in the treatment of human infectious diseases. Besides immunosurveillance in cancer, the pivotal function of immune system is the protection against pathogens, bacteria, viruses and fungi.

As it occurs, even in chronic viral infections, for example, HIV and HBV infection, T cells undergo a continuous lasting antigen exposure, which is the driver of a phenomenon of ‘anergy’ in T cells called T-cell exhaustion. Exhausted CD8 T cells have reduced effector function and poor proliferative capacity and their primary characteristic is the overexpression of inhibitory receptors CTLA4 and PD-1/PD-L1 [10]. The efficacy of monoclonal antibodies against PD-1 and PD-L1, that have notably revolutionized the treatment of cancer, suggests that therapeutically targeting these pathways would also be effective for preventing and treating a range of infectious diseases. Several studies have suggested that ICIs empower T-cell responses giving immunity protection during many chronic viral, bacterial or parasitic infection, including malaria, HIV infection, HBV infection and TB.

In numerous mouse models of cancer and chronic infection, PD-1-targeted therapy suppresses tumor growth and reduces viral load. Similar results have been observed in primates [11]. Many clinical trials on the use of immune checkpoint blockade in chronic viral infection are designed and performed in individuals with malignancies.

Gane *et al.* have recently published a Phase Ib study of Nivolumab (anti PD-1) with and without GS-477, an HBV therapeutic vaccine, in chronic HBV-patients. They observed that Nivolumab was well-tolerated, leading to HBsAg decline in most patients. In one patient they obtained complete HBsAg seroconversion and HBsAg loss [12]. Gay et al. reported the role of an anti-PD-L1 antibody (BMS-936559) in HIV-infected patients in a Phase II study, obtaining an enhance of HIV-1-specific immunity in most of the participants. This is the only trial of an ICI in HIV patients without cancer disease [13]. Interestingly, the inhibition of the immune checkpoint system CD200-CD200R1 has shown positive effects on coronaviruses, restoring IFN production and increasing virus clearance [14,15].

A trial with another ICI anti-PD1, Camrelizumab, is also ongoing in COVID-19 patients.

Although the data from clinical trials in infectious diseases are still weak, checkpoint inhibitors are proving to have great potential in controlling viral infections and reducing viral load. Whereas on one hand the checkpoint blockade can contribute to ‘fuel’ the cytokine storm in case of coexistence of COVID-19, on the other hand, the recovery of immunocompetence could play a protective or even therapeutic role against the viral infection.

We can therefore hypothesize two scenarios:

-  The first ‘pre-infectious phase’: the population treated with ICI is more ‘resistant’ to the attack of COVID-19;
-  The second ‘infectious phase’: patients receiving immunotherapy, when contracting the COVID-19, may develop more serious respiratory symptoms and complications, sustained together by the ‘cytokine storm’ and the ICI-immune mediated injury.

Evidence-based guidelines on the correct management of cancer ICIs during the COVID-19 outbreak are currently lacking and experts are only providing generic practical recommendations [16–19] such as:

-  To delay ICI in case of durable and long-lasting responses, considering the growing evidence of a durable benefit from immunotherapy after treatment outage;
-  To prefer regimens with longer interval, for example, durvalumab 4-weekly administration (instead of 2-weekly), nivolumab 480 mg every 4 weeks or pembrolizumab 400 mg every 6 weeks, in order to reduce the frequency of hospital visits;
-  To delay durvalumab (within 42 days) for III stage lung cancer after concomitant chemoradiotherapy;
-  To postpone immunotherapy in case of flu symptoms, in particular fever, cough, myalgia and fatigue. All presumed cases should be tested with nasopharyngeal swab-PCR for definite diagnosis. If a patient results positive to SARS-CoV-2 and is asymptomatic, 28 days of delay and two negative tests (at 1-week interval) should be considered before restarting the treatment.

The issue whether to continue or delay immunotherapy during the coronavirus outbreak is crucial, since these agents may be detrimental in the case of virus-related pneumonia, but at the same time they are quite effective and potentially curative in cancer, especially in melanoma. A multicentric registry should be strongly encouraged to understand the impact of the COVID-19 infection on cancer patients and in particular on patients undergoing immunotherapy, and to identify the risk factors for poor outcome.

## Conclusion

The COVID-19 pandemic represents a unique challenge but also a learning opportunity. The complex immune responses for patients on immunotherapy treatment against noncancer antigens are largely unknown and giving an incorrect interpretation is a possibility.

ICIs have only minimally been studied in human infectious diseases; therefore, it is not excluded that they may soon revolutionize the treatment against infectious agents, including COVID-19. Unfortunately, in the era of ICIs, the clinical advances in the oncology field are yet to be matched with those in the infectious, but there are broad perspectives for investigations and developments in future.
